# APPLICATION OF QUALITATIVE METHODS IN THE DEVELOPMENT OF AN INFORMANT-REPORTED MEASURE OF LUCIDITY

**DOI:** 10.1093/geroni/igac059.353

**Published:** 2022-12-20

**Authors:** Mildred Ramirez, Jeanne Teresi, Julie Ellis, Paloma Gonzalez-Lopez, Stephanie Silver, Usama Bhatti, Davangere Devanand, Jose Luchsinger

**Affiliations:** Hebrew Home at RiverSpring Health, Riverdale, New York, United States; Research Division, HHAR, Riverdale, New York, United States; La Trobe University, Nursing and Midwifery, Bundoora, Victoria, Australia; Research Division, Hebrew Home at Riverdale by RiverSpring Health, Riverdale, New York, United States; Research Division, Hebrew Home at Riverdale by RiverSpring Health, Riverdale, New York, United States; Research Division, Hebrew Home at Riverdale by RiverSpring Living, Bronx, New York, United States; Columbia University Irving Medical Center, New York, New York, United States; Department of Medicine, Columbia University, New York, New York, United States

## Abstract

The aim was a qualitative evaluation of a lucidity measure for front-line staff or family informants. A sequential approach to item development and evaluation was followed: refinement of the operationalization of the construct; review of seminal items, modification, and purification; confirmation of the feasibility of reporting methodology. Modified focus groups were conducted with 20 staff and 10 family members who participated using a web-based survey. Sample themes included: reaction when hearing the term; words that come to mind; description of and first reaction to referenced or observed “lucidity” events. Data were extracted from Qualtrics for analysis using NVivo. Semi-structured cognitive interviews were conducted with10 health professionals working with older adults with cognitive impairment. An external advisory board reviewed the clarity, breadth, and scope of the conceptual definition and item content. Suggestions for item modification derived from the focus groups and cognitive interviews resulted in the final lucidity measure.

